# A calibration-aware hierarchical CNN-SWIN fusion framework for robust Cross-Dataset brain MRI analysis

**DOI:** 10.3389/fnins.2026.1782306

**Published:** 2026-04-14

**Authors:** Ayesha Younis, Li Qiang, Abdur Rehman, Hamid Hussain, Mohammed Jajere Adamu, Halima Bello Kawuwa

**Affiliations:** 1 School of Microelectronics, Tianjin University, Tianjin, China; 2 Department of Data Science, National University of Computer and Emerging Sciences (FAST–NUCES), Lahore, Pakistan; 3 School of Material Science and Engineering, Zhejiang University, Hangzhou, China; 4 Department of Computer Science, Yobe State University, Damaturu, Nigeria; 5 Center for Distance and Online Education, Lovely Professional University, Phagwara, Punjab, India; 6 Department of Biomedical Engineering, School of Precision Instruments and Opto-electronics Engineering, Tianjin University, Tianjin, China

**Keywords:** brain MRI analysis, calibration-aware learning, CNN-Transformer fusion, Cross-Dataset generalization, hierarchical feature fusion, probabilistic calibration

## Abstract

**Introduction:**

Deep learning approaches have become central to brain MRI analysis; however, their reliability under dataset shift remains a critical barrier to safe and scalable deployment in neuroscience and clinical research. While convolutional neural networks (CNNs) provide strong locality-driven inductive biases for robust feature extraction, they lack global contextual awareness. Conversely, transformer-based architectures capture long-range dependencies but often exhibit reduced robustness and miscalibrated confidence when applied to heterogeneous medical imaging data, particularly in Cross-Dataset settings.

**Methods:**

In this work, we propose a *calibration-aware* hierarchical CNN-Transformer fusion framework designed for robust brain MRI analysis under dataset shift. The architecture integrates a pretrained multi-scale CNN backbone with a hierarchical transformer branch and performs scale-aligned fusion through cross-attention mechanisms. By allowing local convolutional features to selectively query global contextual representations, the proposed design maintains stable feature contributions during fusion and mitigates overconfident reliance on transformer features when generalization degrades across datasets. The framework is evaluated using a strict Cross-Dataset protocol, where models are trained on one dataset and tested on a distinct dataset.

**Results:**

Experimental results demonstrate that the proposed fusion model achieves competitive classification performance while substantially improving probabilistic calibration relative to both CNN-only and transformer-only baselines. Specifically, the model attains an average accuracy of 99.20% and achieves lower Expected Calibration Error (ECE = 0.0041), Brier score (0.0028), and Negative Log-Likelihood (NLL = 0.0277) compared to a standalone Swin Transformer and a strong ResNet50 baseline.

**Discussion:**

These findings demonstrate that calibration-aware hierarchical CNN-Transformer fusion enhances both predictive reliability and robustness under Cross-Dataset evaluation. By improving the alignment between predictive confidence and empirical correctness, the proposed method supports safer large-scale analysis of heterogeneous brain MRI data, with important implications for multi-center neuroscience studies and trustworthy clinical decision support.

## 1 Introduction

Automated analysis of brain magnetic resonance imaging (MRI) plays a central role in modern neuroscience research and clinical workflows, supporting tasks such as disease characterization, population-level studies, and diagnostic decision support. In recent years, deep learning has become the dominant paradigm for brain MRI analysis, largely driven by convolutional neural networks (CNNs) that demonstrate strong inductive biases for capturing local anatomical structures, texture patterns, and spatially coherent features (Litjens et al., 2017; Shen et al., 2017). Architectures such as ResNet (He et al., 2016) have shown notable robustness on limited and heterogeneous medical imaging datasets and continue to serve as strong baselines for a wide range of brain MRI analysis tasks.

Despite their effectiveness, CNNs rely on fixed local receptive fields, which limits their ability to model long-range spatial dependencies and global contextual relationships that may be important for interpreting distributed or diffuse brain abnormalities. To address this limitation, transformer-based architectures have been introduced to computer vision and medical imaging. Vision Transformers and hierarchical variants such as the Swin Transformer (Liu et al., 2021) employ self-attention mechanisms to capture long-range contextual information and have demonstrated promising results across several medical image analysis applications (Zhang et al., 2022). Hybrid CNN-Transformer architectures, including UNETR and SwinUNETR, further explore the integration of convolutional locality with attention-based global modeling, particularly in segmentation-oriented frameworks (Hatamizadeh et al., 2022a,b).

However, accumulating evidence indicates that transformer-based models exhibit important limitations in medical imaging scenarios characterized by limited training data, heterogeneous acquisition protocols, and dataset shift. Prior studies report that transformers may underperform strong CNN baselines in terms of robustness and generalization when evaluated on unseen datasets (Raghu et al., 2021; Li et al., 2022). Moreover, transformer architectures are often prone to overconfident predictions, leading to miscalibrated probability estimates under distribution shift (Ovadia et al., 2019; Müller and Smith, 2022). In brain MRI analysis, such miscalibration is not merely a technical concern: overconfident incorrect predictions can obscure uncertainty and potentially undermine clinical safety by influencing diagnostic interpretation, treatment planning, or downstream research conclusions.

To leverage the complementary strengths of CNNs and transformers, a growing body of work has proposed hybrid fusion architectures that combine convolutional feature extraction with attention-based global modeling (Wang G. et al., 2021; Heidari et al., 2023). While these approaches enhance representational capacity, most are primarily designed to optimize in-domain performance and are typically evaluated under within-dataset validation protocols. In addition, prior CNN-Transformer fusion studies largely focus on segmentation rather than classification and rarely examine how architectural fusion choices affect probabilistic calibration under distribution shift. Consequently, it remains unclear whether existing fusion mechanisms improve confidence reliability when models are deployed across scanners, institutions, and heterogeneous patient populations.

Reliable confidence estimation is particularly critical in safety-aware brain MRI analysis. Under dataset shift, deep neural networks may maintain high accuracy while exhibiting degraded calibration, resulting in a mismatch between predicted confidence and empirical correctness. Such confidence–accuracy gaps can obscure model uncertainty and reduce trustworthiness in clinical or multi-center research settings. Addressing this issue requires not only evaluating accuracy under Cross-Dataset conditions but also explicitly analyzing calibration behavior as a first-class objective.

In this work, we propose a *calibration-aware* hierarchical CNN-Transformer fusion framework designed explicitly to improve confidence reliability under Cross-Dataset distribution shift. The proposed approach integrates a pretrained multi-scale CNN backbone with a hierarchical transformer branch and performs scale-aligned fusion through cross-attention mechanisms. Unlike naive concatenation or additive aggregation strategies, our design allows convolutional features to act as structured queries over transformer representations, preserving locality-driven robustness while adaptively modulating global contextual contributions through learnable fusion weights. This asymmetric interaction stabilizes feature integration when the transformer branch generalizes less reliably across datasets.

Crucially, we evaluate the proposed framework under a strict train-on-one-dataset, test-on-another protocol, without *post-hoc* temperature scaling, ensembling, or target-domain adaptation. This setting isolates the architectural effect of hierarchical fusion on probabilistic calibration and provides a realistic assessment of deployment-relevant robustness. To our knowledge, this is among the first studies to explicitly examine hierarchical CNN-Transformer fusion from a calibration perspective under strict Cross-Dataset evaluation for brain MRI classification.

### 1.1 Contributions

- We propose a hierarchical multi-scale CNN-Transformer fusion architecture that integrates local convolutional features with global transformer representations through scale-aligned cross-attention, explicitly designed for robust brain MRI analysis under dataset shift.
- We demonstrate that structured cross-attention fusion alone—without *post-hoc* temperature scaling or ensembling—can materially improve probabilistic reliability, as measured by Expected Calibration Error (ECE), Brier score, and Negative Log-Likelihood (NLL), under strict Cross-Dataset evaluation.
- We conduct extensive Cross-Dataset experiments on two independent brain MRI datasets using a train-on-one, test-on-another protocol, showing that the proposed method achieves performance comparable to or better than strong CNN baselines while consistently improving confidence calibration relative to both CNN-only and transformer-only models.
- We provide a comprehensive evaluation highlighting the importance of calibration-aware architectural design for reliable brain MRI analysis, with direct implications for multi-center neuroscience research and safety-critical clinical decision support systems.

## 2 Related work

### 2.1 CNN-based brain MRI analysis

Convolutional neural networks (CNNs) remain a strong baseline for brain MRI analysis due to their locality-driven inductive biases, which effectively capture texture, boundaries, and anatomical structure. Transfer learning with ImageNet-pretrained backbones such as ResNet (He et al., 2016) is widely used to stabilize optimization and improve generalization, often yielding strong in-domain performance on curated medical datasets (Litjens et al., 2017; Shen et al., 2017).

However, CNN performance can degrade under acquisition variability arising from differences in scanner vendors, imaging protocols, patient populations, and dataset curation strategies. Such distribution shifts expose limitations of within-dataset validation protocols and motivate explicit Cross-Dataset evaluation. Recent systematic reviews emphasize that robustness to dataset shift remains a primary barrier to safe clinical deployment (Matta et al., 2024).

### 2.2 Vision Transformers and Swin-style models in medical imaging

Vision Transformers (ViT) introduced global self-attention as an alternative to convolutional inductive bias, demonstrating strong scaling behavior in large-data regimes (Dosovitskiy et al., 2021). Data-efficient variants such as DeiT improved performance in limited-data settings through distillation and augmentation (Touvron et al., 2021). The Swin Transformer further extended this paradigm by incorporating hierarchical feature extraction and shifted-window attention, enabling multi-scale contextual modeling with improved computational efficiency (Liu et al., 2021).

In medical imaging, transformer encoders and Swin-style architectures are frequently adopted to capture long-range spatial dependencies, particularly in anatomically complex settings. Hybrid CNN-Transformer designs such as TransUNet and UNETR integrate convolutional feature extraction with attention-based global modeling (Chen et al., 2021; Hatamizadeh et al., 2021). While these architectures demonstrate strong representational capacity, most are developed for segmentation tasks and evaluated primarily under in-domain settings.

### 2.3 CNN-Transformer fusion mechanisms

Fusion architectures aim to combine CNN locality with transformer-based global context through strategies such as feature concatenation, additive aggregation, learned re-weighting, or cross-attention between streams. Cross-attention is particularly appealing because it enables structured interaction between representations, allowing one pathway to query complementary information from another.

Despite these advances, multi-scale fusion remains challenging. Misaligned spatial resolutions, channel mismatches, and unconstrained aggregation weights can destabilize optimization or degrade strong CNN baselines when transformer features generalize poorly under distribution shift. Consequently, effective fusion for brain MRI analysis requires careful feature alignment and controlled interaction rather than naive combination.

### 2.4 Calibration and reliability under dataset shift

For safety-critical applications in neuroscience and clinical decision support, predictive confidence must be reliable in addition to accurate. Modern neural networks are often miscalibrated, motivating explicit evaluation using metrics such as Expected Calibration Error (ECE), Brier score, and Negative Log-Likelihood (NLL) (Guo et al., 2017). Importantly, calibration can deteriorate substantially under dataset shift even when accuracy remains high, resulting in overconfident incorrect predictions (Ovadia et al., 2019).

Existing calibration strategies commonly rely on *post-hoc* techniques such as temperature scaling, Platt scaling, ensembling, or Monte Carlo dropout. Although these methods can improve confidence alignment, they operate independently of architectural design and do not directly address the feature interaction mechanisms that may contribute to miscalibration. Recent work further highlights the need for robustness-aware evaluation protocols that assess reliability under deployment-like conditions (Park et al., 2021).

### 2.5 Gap analysis

While CNN-Transformer fusion has been widely explored to enhance representational capacity and in-domain performance—particularly in segmentation—its impact on probabilistic calibration under strict Cross-Dataset evaluation remains insufficiently examined for brain MRI classification. Most prior studies report within-dataset accuracy and do not analyze how fusion design influences confidence behavior when models are applied to unseen scanners or institutions.

Addressing this gap requires (i) evaluating fusion mechanisms under train-on-one, test-on-another protocols and (ii) explicitly measuring calibration alongside accuracy. This need motivates the calibration-aware hierarchical CNN-Transformer fusion framework proposed in this work.

## 3 Proposed method

This section presents the proposed *Hierarchical Multi-Scale CNN-Transformer Fusion Framework*, designed as a calibration-aware brain MRI analysis method robust to dataset shift. The design is motivated by two complementary observations. First, convolutional neural networks (CNNs) are highly effective at capturing fine-grained local structures such as boundaries, intensity discontinuities, and texture variations that are critical for reliable brain MRI interpretation. Second, transformer-based architectures, and in particular the Swin Transformer, provide powerful mechanisms for modeling long-range spatial dependencies and global anatomical context that are difficult to encode using fixed convolutional receptive fields alone. However, when applied independently, both paradigms exhibit limitations under Cross-Dataset evaluation: CNNs may lack sufficient global contextual awareness, while transformers may underperform on small or subtle regions due to token aggregation and hierarchical downsampling effects.

**Figure 1:**
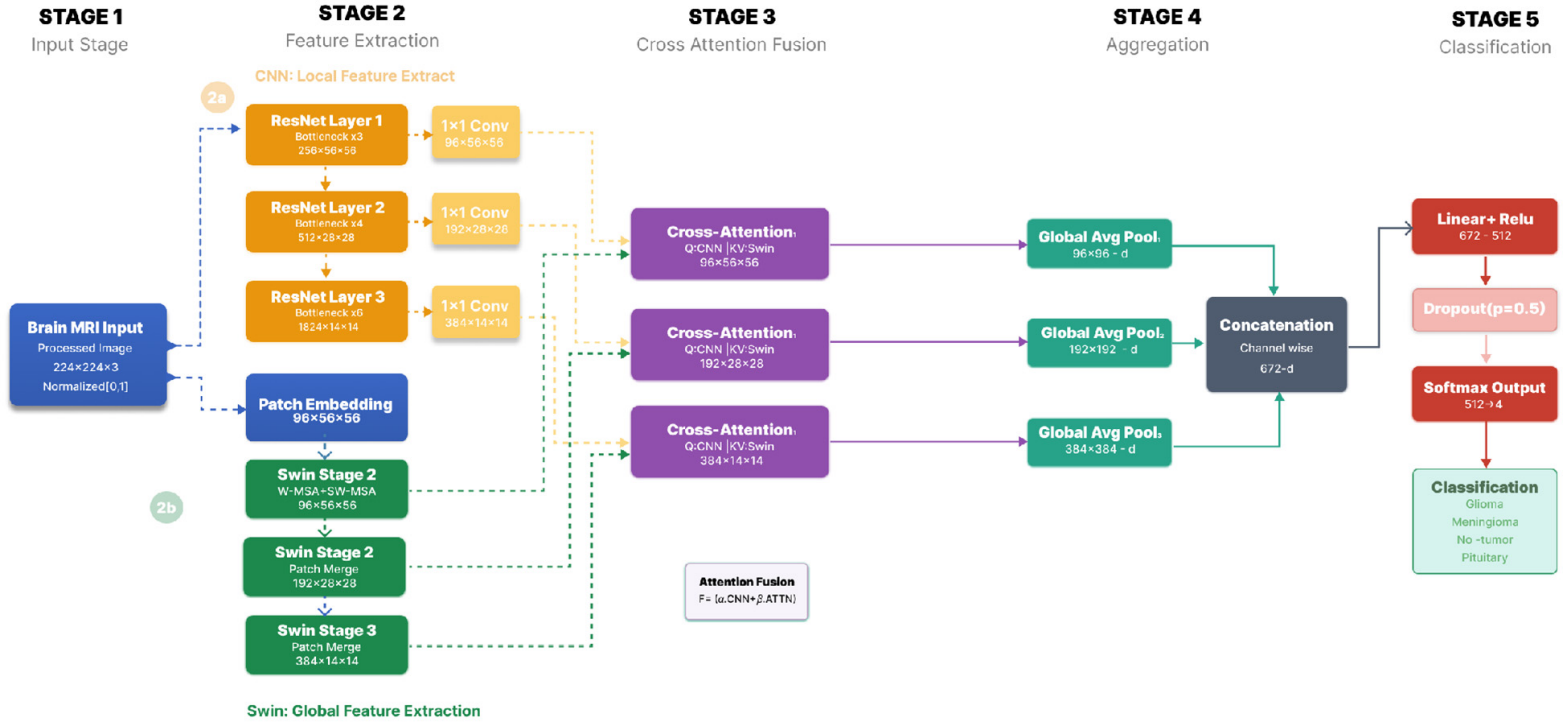
Overview of the proposed hierarchical CNN–Swin fusion framework. A multi-scale ResNet-based CNN branch extracts local texture and boundary information, while a pretrained Swin Transformer branch models global contextual dependencies. At each resolution, cross-attention fusion aligns CNN queries with Swin keys and values. The fused representations are globally pooled, concatenated, and classified by a lightweight MLP head.

To address these challenges, we propose a unified framework that explicitly integrates local and global representations through *hierarchical cross-attention fusion*. The network consists of two parallel feature extraction branches—a multi-scale CNN encoder and a pretrained Swin Transformer encoder—whose outputs are fused at three spatial resolutions. At each scale, CNN features act as queries that selectively attend to transformer-derived contextual features, allowing local evidence to be refined using global anatomical cues while preserving locality-driven robustness. The resulting multi-scale fused representations are aggregated and passed to a lightweight classification head. An overview of the complete pipeline is illustrated in Figure 1.

Formally, given an input MRI slice *x*, the overall mapping implemented by the model can be summarized as:


$$F(x):x\overset{\text{Features}}{\to }\;{\left\{C_{i},S_{i}\right\}}_{i=1}^{3}\overset{\text{Fusion}}{\to }M_{fuse}\;\overset{\text{Agg.}}{\to }\text{Softmax}(z)=\hat{y}$$ (1)

Where **C**_*i*_ and **S**_*i*_ represent the *i*-th scale CNN and Swin features respectively, **M**_*fuse*_ is the fused multi-scale representation, and ŷ denotes the predicted class probability distribution.

**Notation**. An input MRI slice is denoted by *x*∈ℝ^3 × *H*×*W*^. For fusion scale *s*∈{1, 2, 3}, the CNN feature map is $F_{s}^{\text{cnn}}\in {\mathbb{R}}^{C_{s}\times H_{s}\times W_{s}}$, the Swin Transformer feature map is $F_{s}^{\text{swin}}\in {\mathbb{R}}^{D_{s}\times {Ĥ}_{s}\times {Ŵ}_{s}}$, and the fused representation is $F_{s}^{\text{fuse}}\in {\mathbb{R}}^{C_{s}\times H_{s}\times W_{s}}$. In our implementation, (*C*_1_, *C*_2_, *C*_3_) = (96, 192, 384) with spatial resolutions (56, 56), (28, 28), and (14, 14), respectively. The mathematical formulation of the proposed framework is presented in Equations 1–22.

### 3.1 Multi-scale CNN encoder

The CNN branch is designed to capture local and mid-level tumor characteristics that are essential for reliable diagnosis, including boundary sharpness, texture irregularities, and local contrast variations. We adopt a ResNet50 backbone and extract feature maps from three intermediate stages corresponding to progressively increasing receptive fields. Let *E*_cnn_(·) denote the CNN encoder. Given input *x*, the encoder produces:


$$\begin{array}{l} \left(F_{1}^{\text{raw}},F_{2}^{\text{raw}},F_{3}^{\text{raw}}\right)=E_{\text{cnn}}\left(x\right), \end{array}$$ (2)

where $F_{1}^{\text{raw}}\in {\mathbb{R}}^{256\times 56\times 56}$, $F_{2}^{\text{raw}}\in {\mathbb{R}}^{512\times 28\times 28}$, and $F_{3}^{\text{raw}}\in {\mathbb{R}}^{1024\times 14\times 14}$.

To ensure compatibility with transformer features during fusion, each stage is projected using a 1 × 1 convolution:


$$\begin{array}{l} F_{s}^{\text{cnn}}=\phi_{s}\left(F_{s}^{\text{raw}}\right),\;\phi_{s}\left(\cdot \right)=W_{1\times 1}^{\left(s\right)}*\left(\cdot \right), \end{array}$$ (3)

resulting in channel dimensions 96, 192, and 384 at the three respective scales. These projections preserve spatial structure while aligning feature dimensions for cross-attention fusion.

### 3.2 Pretrained Swin Transformer encoder

To complement convolutional locality, the second branch employs a pretrained Swin Transformer, which provides hierarchical global context modeling through shifted-window self-attention. We use a Swin-T backbone accessed via the timm library with features_only=True, enabling extraction of intermediate representations.

Given input *x*, the transformer encoder produces:


$$\begin{array}{l} \left({\tilde{F}}_{1}^{\text{swin}},{\tilde{F}}_{2}^{\text{swin}},{\tilde{F}}_{3}^{\text{swin}}\right)=E_{\text{swin}}\left(x\right), \end{array}$$ (4)

corresponding to three hierarchical stages. To ensure dimensional compatibility, we apply optional 1 × 1 projections:


$$\begin{array}{l} F_{s}^{\text{swin}}=\psi_{s}\left({\tilde{F}}_{s}^{\text{swin}}\right), \end{array}$$ (5)

where ψ_*s*_ is either an identity mapping or a learned projection to (96, 192, 384). These features serve as the global contextual source during fusion.

### 3.3 Hierarchical cross-attention fusion

A key component of the proposed method is a hierarchical cross-attention fusion mechanism that integrates local CNN features with global transformer representations in a spatially aligned manner. Rather than naïvely concatenating or summing features, the CNN pathway is used to *query* transformer features at each spatial location, enabling structured interaction between local tumor evidence and global anatomical context.

To enforce spatial alignment, the Swin feature maps are resized to match CNN resolutions using bilinear interpolation:


$$\begin{array}{l} {\bar{F}}_{s}^{\text{swin}}=\text{Interp}\left(F_{s}^{\text{swin}},\left(H_{s},W_{s}\right)\right). \end{array}$$ (6)

Queries, keys, and values are generated using 1 × 1 convolutions:


$$\begin{array}{l} Q_{s}=W_{s}^{Q}*F_{s}^{\text{cnn}},\;K_{s}=W_{s}^{K}*{\bar{F}}_{s}^{\text{swin}},\;V_{s}=W_{s}^{V}*{\bar{F}}_{s}^{\text{swin}}. \end{array}$$ (7)

The tensors are reshaped into *h* = 8 attention heads with per-head dimensionality *d*_*s*_ = *C*_*s*_/*h* and flattened into *N*_*s*_ = *H*_*s*_*W*_*s*_ tokens. Cross-attention is computed via scaled dot-product attention:


$$\begin{array}{l} A_{s}=\text{Softmax}\left(\frac{Q_{s}K_{s}^{⊤}}{\sqrt{d_{s}}}\right),\;O_{s}=A_{s}V_{s}. \end{array}$$ (8)

After merging attention heads and applying a 1 × 1 output projection, the resulting feature map is denoted ${Ô}_{s}\in {\mathbb{R}}^{C_{s}\times H_{s}\times W_{s}}$.

Finally, CNN and attention-derived features are combined using a residual-style weighted sum:


$$\begin{array}{l} F_{s}^{\text{fuse}}=\alpha_{s}F_{s}^{\text{cnn}}+\beta_{s}{Ô}_{s}, \end{array}$$ (9)

In practice, α_*s*_ is initialized to 1.0 and β_*s*_ to 0.1, biasing early training toward the CNN pathway.

where α_*s*_ and β_*s*_ are scalar learnable parameters initialized to favor the CNN pathway and optimized jointly with the cross-attention weights. Unlike static residual or additive fusion, these parameters are learned in conjunction with scale-specific cross-attention, enabling adaptive modulation of global contextual contribution under dataset shift. This design preserves strong locality-driven CNN representations while selectively incorporating transformer-derived context, particularly when transformer features generalize less reliably across datasets. As a result, the fusion mechanism mitigates overconfident reliance on global context and contributes directly to improved probabilistic calibration, as demonstrated in

This initialization encodes an inductive bias favoring local evidence early in training, which we hypothesize contributes to improved calibration under distribution shift (Section 5).

### 3.4 Multi-scale aggregation and classification

The fused representations capture complementary information at multiple resolutions. Global average pooling is applied at each scale:


$$\begin{array}{l} z_{s}=\text{GAP}\left(F_{s}^{\text{fuse}}\right),\;s\in \left\{1,2,3\right\}. \end{array}$$ (10)

The pooled vectors are concatenated to form a unified embedding:


$$\begin{array}{l} z=\left[z_{1}||z_{2}||z_{3}\right]\in {\mathbb{R}}^{672}. \end{array}$$ (11)

Classification is performed using a lightweight two-layer MLP with ReLU activation and dropout:


$$\begin{array}{l} h=\text{ReLU}\left(W_{1}z+b_{1}\right),\;ŷ=\text{Softmax}\left(W_{2}\text{Dropout}\left(h\right)+b_{2}\right), \end{array}$$ (12)

where ŷ denotes the predicted probability distribution over tumor classes. The network is trained end-to-end using the standard cross-entropy loss:


$$\begin{array}{l} L_{\text{CE}}=-\overset{C}{\underset{c=1}{\sum }}y_{c}\log\left({ŷ}_{c}\right). \end{array}$$ (13)

By integrating convolutional locality and transformer-based global reasoning through hierarchical cross-attention, the proposed framework constructs a unified representation that is both discriminative and robust under Cross-Dataset evaluation. Unlike prior CNN-Transformer fusion approaches that primarily optimize in-domain accuracy, this design explicitly targets stable feature interaction and confidence reliability under dataset shift, laying the foundation for the improved accuracy and calibration reported in Section 5.

## 4 Dataset and experimental setup

**Figure 2:**
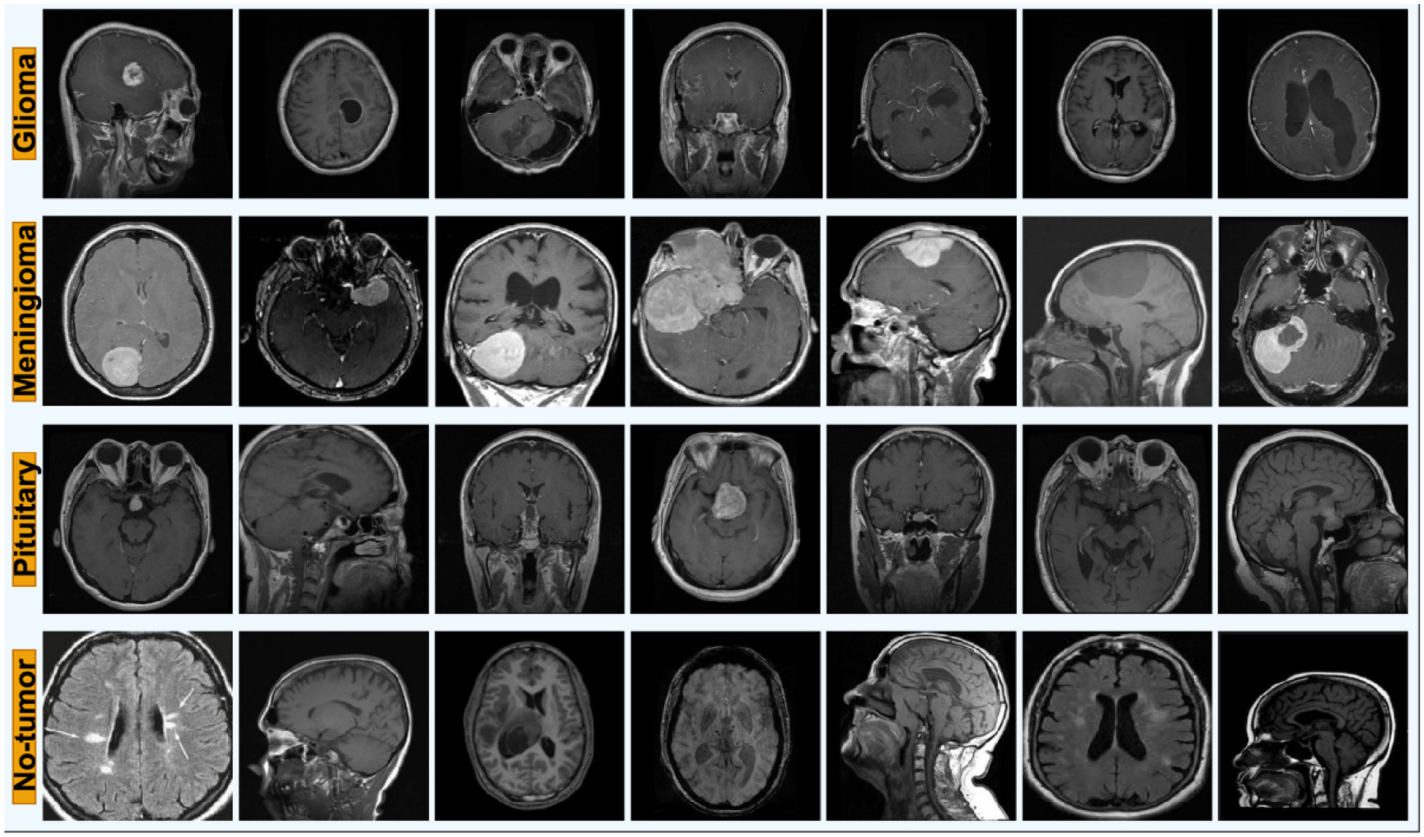
Representative samples across the four classes.

To rigorously evaluate Cross-Dataset generalization and confidence reliability, we conduct experiments on two publicly available brain MRI classification datasets collected from different sources, acquisition protocols, and scanner environments. Using heterogeneous datasets provides a deployment-relevant assessment of distribution shift, which is essential for methodological validation in brain imaging studies and for safety-critical clinical decision support. Representative MRI samples across the four diagnostic categories are shown in Figure 2, highlighting visual differences between glioma, meningioma, pituitary, and no-tumor classes.

**Figure 3:**
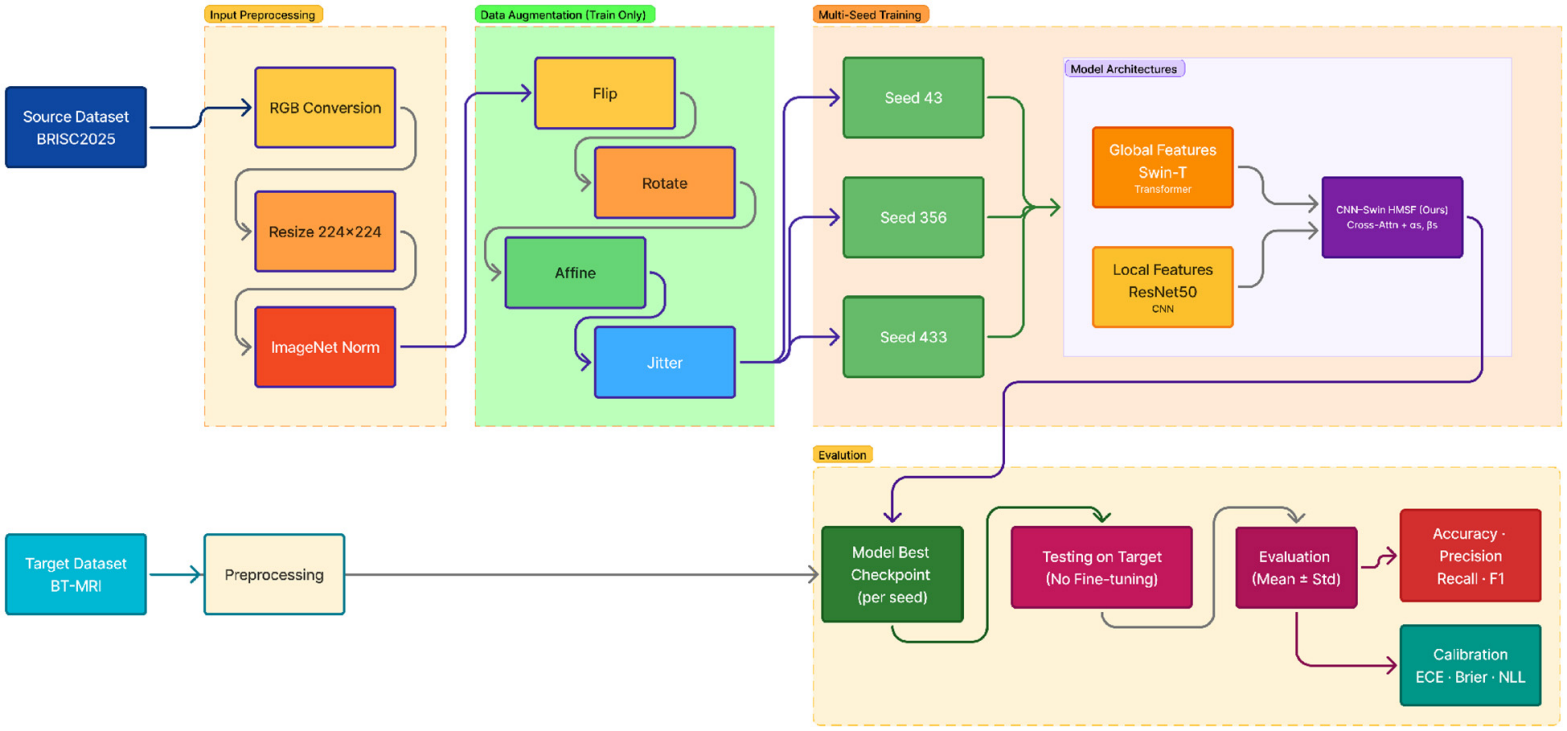
End-to-end pipeline for cross-dataset MRI classification, including preprocessing, multi-model feature extraction, prediction, and calibrated evaluation.

Specifically, we adopt a strict *Cross-Dataset evaluation protocol* in which the model is trained on one dataset and evaluated on a separate dataset *without* any target-dataset validation, or data leakage. This setting reflects realistic scenarios in which labeled data from a target hospital or scanner is unavailable, and it directly tests whether predictive confidence remains meaningful under heterogeneity. An overview of the complete training, preprocessing, multi-seed learning, and Cross-Dataset evaluation pipeline is illustrated in Figure 3.

### 4.1 BRISC2025 dataset

The BRISC2025 (Fateh et al., 2025) dataset is a large-scale, curated collection of T1-weighted contrast-enhanced brain MRI slices designed for multi-class classification. It contains four diagnostic categories: *glioma, meningioma, pituitary adenoma*, and *no-tumor*. Images originate from multiple clinical sources, introducing natural variability in intensity distributions, tumor morphology, and background composition.

**Figure 4:**
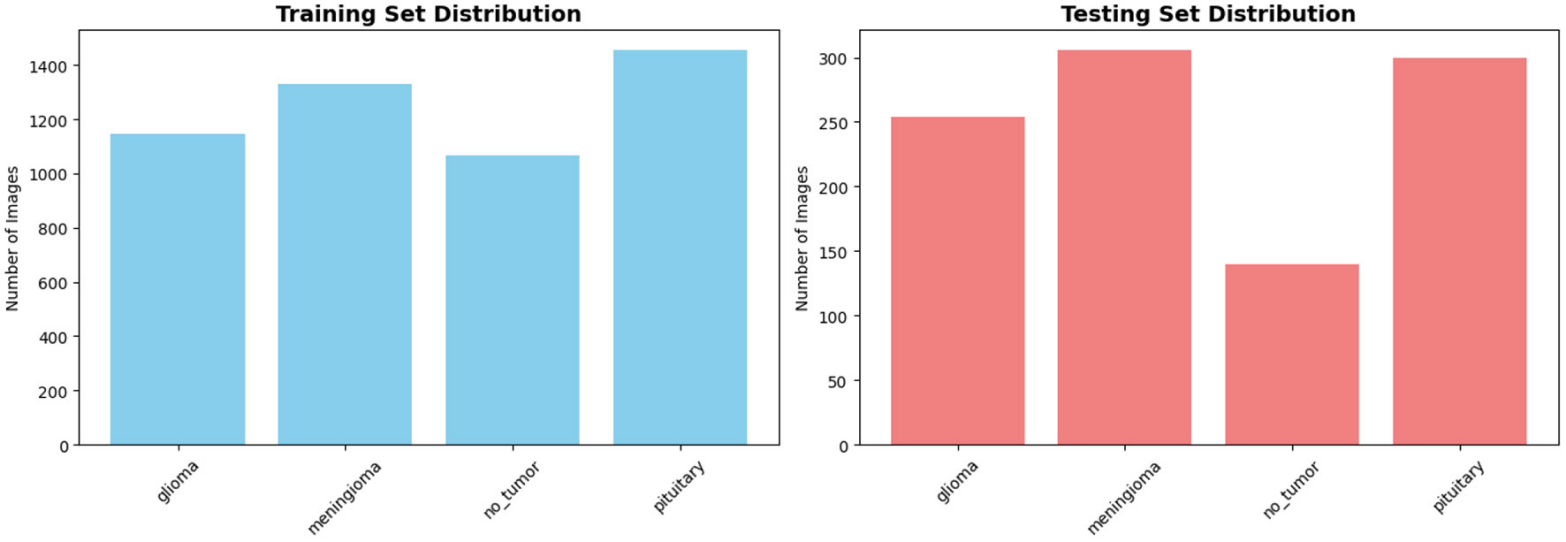
Class-wise distribution of the BRISC2025 dataset across training and testing splits.

The dataset is provided with predefined training and testing splits. Table 1 summarizes the class-wise distribution used in our experiments, while Figure 4 illustrates the corresponding distribution across training and testing sets.

**Table 1:** Class-wise distribution of the BRISC2025 dataset.

Class	Training	Testing	Total
Glioma	1,147	254	1,401
Meningioma	1,329	306	1,635
No-tumor	1,067	140	1,207
Pituitary	1,457	300	1,757
**Total**	**5,000**	**1,000**	**6,000**

Bold values indicate best-performing results across models.

The BRISC2025 dataset exhibits moderate class imbalance, particularly between *no-tumor* and *pituitary* cases, making it suitable for evaluating both discriminative performance and calibration behavior under uneven class priors.

### 4.2 BT-MRI dataset

The BT-MRI dataset is a widely used benchmark for brain MRI classification, comprising contrast-enhanced MRI slices collected from multiple institutions. It contains the same four diagnostic categories as BRISC2025: *glioma, meningioma, pituitary adenoma*, and *no-tumor*. However, the dataset differs substantially in image appearance, scanner characteristics, background composition, and tumor presentation, making it challenging in Cross-Dataset evaluation.

**Figure 5:**
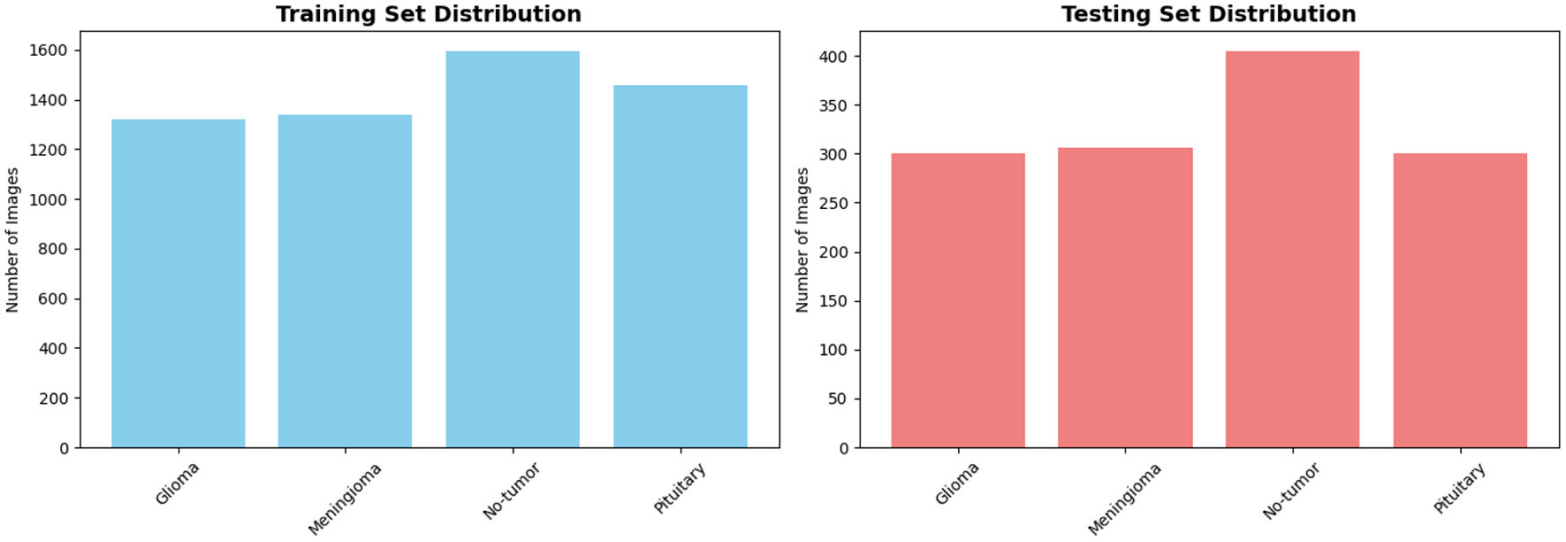
Class-wise distribution of the BT-MRI dataset across training and testing splits.

The dataset is released with predefined training and testing partitions. The class-wise statistics are reported in Table 2, while Figure 5 provides a visual representation of the class distribution across training and testing splits. Compared to BRISC2025, BT-MRI contains a higher proportion of *no-tumor* samples and exhibits different intensity and background characteristics. These discrepancies induce a non-trivial data shift that challenges both model generalization and probability calibration.

**Table 2:** Class-wise distribution of the BT-MRI dataset.

Class	Training	Testing	Total
Glioma	1,321	300	1,621
Meningioma	1,339	306	1,645
No-tumor	1,595	405	2,000
Pituitary	1,457	300	1,757
**Total**	**5,712**	**1,311**	**7,023**

Bold values indicate best-performing results across models.

### 4.3 Cross-Dataset evaluation protocol

To assess robustness under deployment-relevant conditions, we adopt a **one-way Cross-Dataset evaluation** strategy. Unless otherwise stated, the model is trained exclusively on the BRISC2025 training set and evaluated on the BT-MRI test set:


$$D_{\text{train}}=\text{BRISC2025},\;D_{\text{test}}=\text{BT-MRI}.$$

No samples from the target dataset are used during training, validation, hyperparameter tuning, or early stopping. This strict separation ensures that all reported results reflect genuine generalization rather than dataset-specific adaptation.

The reverse setting (BT-MRI → BRISC2025) is used only for supplementary analysis and is not included in the main results to maintain clarity of presentation.

Let $D_{S}={\left\{\left(x_{i},y_{i}\right)\right\}}_{i=1}^{N_{S}}$ denote the source dataset and $D_{T}={\left\{\left(x_{j},y_{j}\right)\right\}}_{j=1}^{N_{T}}$ the target dataset, with potentially different joint distributions:


$$\begin{array}{l} p_{S}\left(x,y\right)\neq p_{T}\left(x,y\right). \end{array}$$ (14)

The model parameters θ are optimized by minimizing the empirical risk on $D_{S}$:


$$\begin{array}{l} \theta^{*}=\arg\underset{\theta }{\min}{𝔼}_{\left(x,y\right)\sim D_{S}}\left[L\left(f_{\theta }\left(x\right),y\right)\right], \end{array}$$ (15)

while all reported performance metrics are computed exclusively on $D_{T}$. This protocol prevents data leakage and provides a realistic assessment of robustness and confidence reliability under dataset shift.

This study evaluates methodological robustness using retrospective public datasets. While results are encouraging, prospective clinical validation and multi-site testing are required prior to deployment in real-world diagnostic workflows.

We prioritize the BRISC2025 → BT-MRI direction in the main paper due to its stronger distribution shift characteristics, while reporting the reverse setting in the supplementary material for completeness.

### 4.4 Dataset curation and quality control

To enhance transparency and reproducibility, we explicitly describe the dataset handling and quality control procedures adopted in this study.

Both BRISC2025 and BT-MRI are publicly released datasets with predefined training and testing splits and expert-provided diagnostic labels. No relabeling, class merging, or modification of diagnostic categories was performed. All labels were used exactly as provided by the original dataset creators.

Prior to training, all image files were subjected to automated integrity checks to remove corrupted or unreadable files. We verified consistency of image dimensions and ensured that class labels were correctly mapped. Duplicate file names and visually identical samples were screened to avoid unintended data leakage.

No additional filtering based on tumor size, anatomical location, or visual characteristics was applied. All eligible slices within the official dataset partitions were retained to preserve the natural variability and class imbalance properties of each dataset.

Original dataset splits were strictly preserved. In the Cross-Dataset setting, the target dataset was not used for model selection, hyperparameter tuning, or early stopping, ensuring complete separation between training and evaluation data.

These procedures ensure that the reported results reflect genuine Cross-Dataset generalization rather than artifacts introduced by implicit filtering, relabeling, or partition modification.

### 4.5 Preprocessing and data augmentation

To ensure consistency with ImageNet-pretrained backbones and reduce scanner-induced variability, all MRI slices are converted to three-channel RGB format and resized to 224 × 224 pixels.

#### 4.5.1 Normalization

Pixel intensities are normalized using ImageNet mean and standard deviation:


$$\begin{array}{l} I_{\text{norm}}=\frac{I-\mu_{\text{ImageNet}}}{\sigma_{\text{ImageNet}}}, \end{array}$$ (16)

ensuring compatibility with pretrained CNN and Swin Transformer weights.

#### 4.5.2 Data augmentation

During training, we apply stochastic augmentations to improve robustness to appearance variations: random horizontal flipping, small-angle rotations (±15°), affine transformations (scaling and translation), and mild color jitter. These augmentations preserve anatomical plausibility while exposing the model to both local texture perturbations and global structural changes. No augmentation is applied during testing.

### 4.6 Training configuration

All models are implemented in PyTorch and trained using the AdamW optimizer. The CNN branch is initialized with ImageNet-pretrained ResNet50 weights, and the transformer branch uses a pretrained Swin-T backbone obtained via the timm library. Fusion modules and classification heads are trained from scratch.

To account for optimization stochasticity, each experiment is repeated using three independent random seeds:


{43,356,433}.

Results are reported as mean and standard deviation across seeds. Given the limited number of random seeds, results are interpreted primarily through consistency and effect size rather than formal hypothesis testing.

Learning rate scheduling is performed using ReduceLROnPlateau based on validation accuracy on the source dataset. The checkpoint with the highest validation accuracy is retained for final Cross-Dataset evaluation, with the complete training configuration detailed in Table 3.

**Table 3:** Training hyperparameters used in all experiments.

Parameter	Value
Optimizer	AdamW
Initial learning rate	1 × 10^−4^
Weight decay	1 × 10^−2^
Batch size	16
Number of epochs	50
LR scheduler	ReduceLROnPlateau
Scheduler patience	5 epochs
Dropout (classifier)	0.5
Image resolution	224 × 224
Random seeds	43, 356, 433

### 4.7 Evaluation metrics

Model performance is evaluated using both classification accuracy metrics and probabilistic calibration measures, reflecting the dual requirements of discriminative power and confidence reliability in medical diagnosis.

#### 4.7.1 Classification metrics

To assess discriminative performance, we employ Overall Accuracy, as well as macro-averaged Precision, Recall, and F1-score to account for potential class imbalances in the MRI datasets. These are defined based on True Positives (*TP*_*c*_), False Positives (*FP*_*c*_), and False Negatives (*FN*_*c*_) for each class *c*∈{1, …, *C*}:


$$\begin{array}{l} \text{Precision}=\frac{1}{C}\overset{C}{\underset{c=1}{\sum }}\frac{TP_{c}}{TP_{c}+FP_{c}}, \end{array}$$

$$\begin{array}{l} \text{Recall}=\frac{1}{C}\overset{C}{\underset{c=1}{\sum }}\frac{TP_{c}}{TP_{c}+FN_{c}} \end{array}$$ (17)

$$\begin{array}{l} \text{F1-score}=\frac{1}{C}\overset{C}{\underset{c=1}{\sum }}\frac{2\times {\text{Precision}}_{c}\times {\text{Recall}}_{c}}{{\text{Precision}}_{c}+{\text{Recall}}_{c}} \end{array}$$ (18)

Overall accuracy is defined as:


$$\begin{array}{l} \text{Accuracy}=\frac{1}{N_{T}}\overset{N_{T}}{\underset{i=1}{\sum }}𝕀\left({ŷ}_{i}=y_{i}\right), \end{array}$$ (19)

where ${ŷ}_{i}=\arg\underset{c}{\max}p_{\theta }\left(y=c|x_{i}\right)$.

#### 4.7.2 Expected Calibration Error (ECE)

ECE quantifies the mismatch between a model's predicted confidence and its empirical accuracy by discretizing predictions into *M* disjoint bins ${\left\{B_{m}\right\}}_{m=1}^{M}$ based on confidence (Guo et al., 2017). It is defined as:


$$\begin{array}{l} \text{ECE}=\overset{M}{\underset{m=1}{\sum }}\frac{\left|B_{m}\right|}{N_{T}}|\text{acc}\left(B_{m}\right)-\text{conf}\left(B_{m}\right)|, \end{array}$$ (20)

where acc(*B*_*m*_) and conf(*B*_*m*_) represent the accuracy and average confidence within bin *m*, respectively. Under Cross-Dataset evaluation, distributional shifts frequently cause deep models to become systematically overconfident, leading to low accuracy-high confidence failure modes (Lakshminarayanan et al., 2017).

#### 4.7.3 Brier score

The Brier score is a strictly proper scoring rule that evaluates the mean squared error between predicted probabilities and one-hot ground-truth labels (Brier, 1950):


$$\begin{array}{l} \text{Brier}=\frac{1}{N_{T}}\overset{N_{T}}{\underset{i=1}{\sum }}\overset{C}{\underset{c=1}{\sum }}{\left(p_{\theta }\left(y=c|x_{i}\right)-𝕀\left(y_{i}=c\right)\right)}^{2}. \end{array}$$ (21)

Unlike ECE, the Brier score captures both calibration and “sharpness” without requiring binning, making it a robust measure for comparing models under dataset shift (Minderer et al., 2021).

##### 4.7.3.1 Negative Log-Likelihood (NLL)

NLL measures the average log-probability assigned to the correct class:


$$\begin{array}{l} \text{NLL}=-\frac{1}{N_{T}}\overset{N_{T}}{\underset{i=1}{\sum }}\log p_{\theta }\left(y=y_{i}|x_{i}\right). \end{array}$$ (22)

NLL is highly sensitive to miscalibration, as it assigns an unbounded penalty to overconfident incorrect predictions (Ovadia et al., 2019). Improved NLL directly reflects enhanced robustness to the deceptive confidence often seen in standalone Transformer architectures.

Together, ECE, Brier score, and NLL provide complementary perspectives on probabilistic reliability. While ECE offers an interpretable, bin-based calibration diagnostic, the Brier score and NLL provide distribution-level and sample-level assessments, respectively. Reporting all three metrics enables a comprehensive evaluation of calibration behavior under distribution shift, where accuracy alone is insufficient to assess clinical safety.

In this work, these metrics are reported alongside standard classification measures to demonstrate that the proposed CNN-Transformer fusion not only improves classification performance but also enhances confidence reliability when trained on one dataset and tested on another. Although ECE is bin-dependent, reporting it alongside strictly proper scoring rules (Brier score and NLL) ensures that calibration improvements are not artifacts of binning choices.

### 4.8 Implementation details

All experiments are conducted on an NVIDIA A100 GPU with 40 GB memory, using an input resolution of 224 × 224 for all models. The fusion model incurs a modest computational overhead relative to standalone CNN and Swin baselines due to multi-scale cross-attention, while remaining tractable for clinical-scale inference.

All baselines (CNN-only and Swin-only) share the same preprocessing, training configuration, and evaluation protocol described above, ensuring that observed performance differences are attributable to architectural design rather than experimental confounds.

## 5 Results and discussion

This section presents a comprehensive evaluation of the proposed hierarchical CNN-Transformer fusion framework under a strict Cross-Dataset setting. All models are trained exclusively on the source dataset and evaluated on a disjoint target dataset, ensuring that reported results reflect genuine generalization under dataset shift.

To assess whether gains arise from the proposed hierarchical fusion design—as opposed to simply combining two backbones—we include two fusion baselines. Additive fusion directly sums CNN and transformer features without scale alignment or attention and exhibits degraded Cross-Dataset performance, implying that naive aggregation is insufficient under dataset shift. Feature concatenation improves over the standalone Swin Transformer but remains inferior to a strong ResNet50 baseline, further indicating that effective CNN-Transformer fusion requires controlled feature interaction. In contrast, the proposed HMSF consistently outperforms all baselines, supporting the value of scale-aligned cross-attention for robust brain MRI analysis. Unless otherwise stated, results are reported as mean ± standard deviation over three independent runs with random seeds {43, 356, 433}. Given the limited number of seeds, conclusions are drawn primarily from consistency trends and effect sizes rather than formal hypothesis testing.

### 5.1 Cross-Dataset classification performance

Table 4 summarizes classification performance under Cross-Dataset evaluation. The standalone Swin Transformer exhibits substantial performance degradation (accuracy 89.60%), highlighting its sensitivity to distribution shifts. In contrast, the ResNet50 baseline maintains strong performance, consistent with the robustness of convolutional inductive biases under MRI appearance variation.

**Table 4:** Cross-Dataset classification performance (train on source dataset, test on target dataset).

Model	Accuracy	Precision	Recall	F1-score
Swin Transformer	89.60 ± 1.56	0.9047 ± 0.0130	0.8960 ± 0.0156	0.8948 ± 0.0173
CNN–Swin (additive fusion)	93.42 ± 1.21	0.9315 ± 0.0104	0.9289 ± 0.0116	0.9292 ± 0.0109
CNN–Swin (concat fusion)	97.85 ± 0.58	0.9789 ± 0.0051	0.9781 ± 0.0056	0.9782 ± 0.0053
ResNet50	99.07 ± 0.35	0.9908 ± 0.0034	0.9907 ± 0.0035	0.9907 ± 0.0035
**CNN–Swin with HMSF (ours)**	**99.20 ± 0.26**	**0.9921 ± 0.0026**	**0.9920 ± 0.0026**	**0.9920 ± 0.0026**

Results are reported as mean ± standard deviation over three random seeds. Bold values indicate best-performing results across models.

The proposed CNN-Swin fusion achieves the best overall performance, with an average accuracy of **99.20%**, outperforming both the transformer-only baseline and the two fusion baselines, and slightly exceeding ResNet50. While the improvement over ResNet50 is numerically modest, it is consistent across seeds and is accompanied by clearer gains in calibration and confidence reliability (Section 5.4), which are critical for safety-aware deployment under dataset shift.

Across the three random seeds, HMSF consistently matches or exceeds the strongest CNN baseline in accuracy while yielding improved calibration metrics (Section 5.4). A larger-scale study with additional seeds and sites is a natural extension for future work.

These results suggest that hierarchical cross-attention fusion preserves the local discriminative strength of CNNs while selectively incorporating global context from transformers, improving robustness under dataset shift.

**Figure 6:**
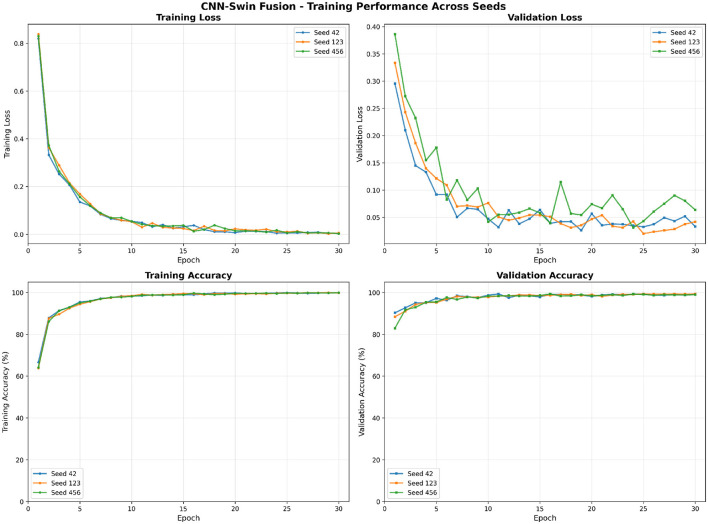
Training and validation dynamics of the proposed CNN-Swin fusion model across three random seeds. **Top**: training loss (left) and validation loss (right). **Bottom**: training accuracy (left) and validation accuracy (right). Consistent convergence and low variance across seeds indicate stable optimization and reproducible learning behavior.

Figure 6 shows that the proposed CNN-Swin fusion model converges consistently across random seeds. Training and validation curves closely align, indicating stable optimization under the reported protocol, with no evidence of severe overfitting. The low variance across runs supports that improvements are not driven by a single favorable initialization.

### 5.2 Confusion matrix analysis

**Figure 7:**
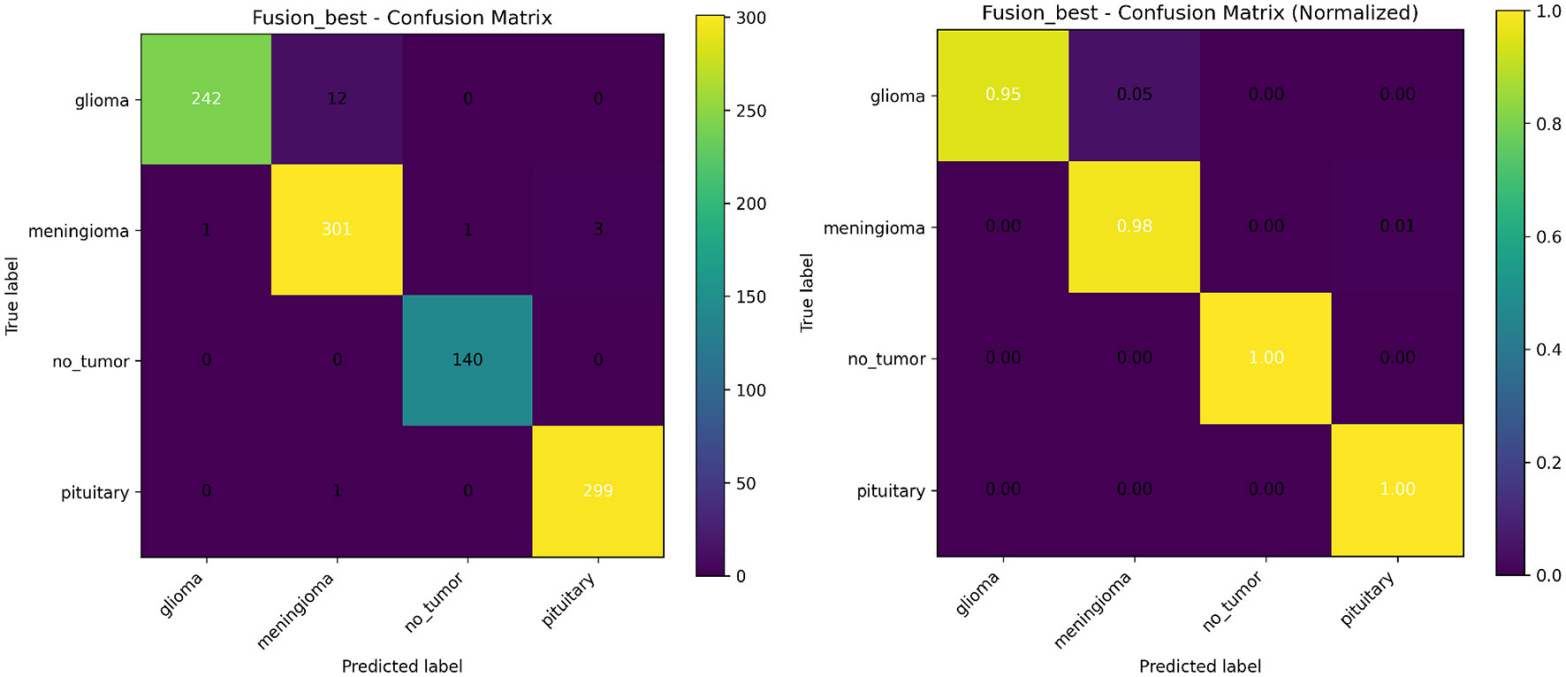
Raw **(left)** and normalized **(right)** confusion matrices for the proposed CNN-Swin fusion model under Cross-Dataset evaluation.

To rigorously assess the categorical robustness of the proposed framework, Figure 7 illustrates the raw and normalized confusion matrices on the target (OOD) dataset. The empirical results reveal a high degree of linear separability in the fused feature space, despite the significant distributional shift. Notably, the model achieves near-optimal sensitivity for glioma and meningioma, categories that frequently exhibit morphological isomorphism in T1-weighted sequences. The mitigation of inter-class leakage between these categories suggests that the scale-aligned cross-attention mechanism successfully disentangles local hyper-intense textural signatures (captured by the CNN) from the broader anatomical context of the dural or intra-axial surroundings (modeled by the Swin Transformer).

The normalized confusion matrix further elucidates the model's class-conditional calibration. By maintaining a balanced diagonal under shift, the fusion architecture demonstrates that its learned scalar weights (α, β) effectively regularize the predictive entropy, preventing the “confidence-accuracy gap” typically observed in standalone transformers. This balanced performance implies that the hierarchical aggregation of multi-scale features acts as a structural prior, enforcing a more conservative and reliable decision boundary. Such reliability is a prerequisite for clinical integration, as it ensures that the reduction in Expected Calibration Error (ECE) is not merely an aggregate statistical artifact but a consistent improvement across diverse tumor etiologies.

### 5.3 ROC and Precision–Recall curves

**Figure 8:**
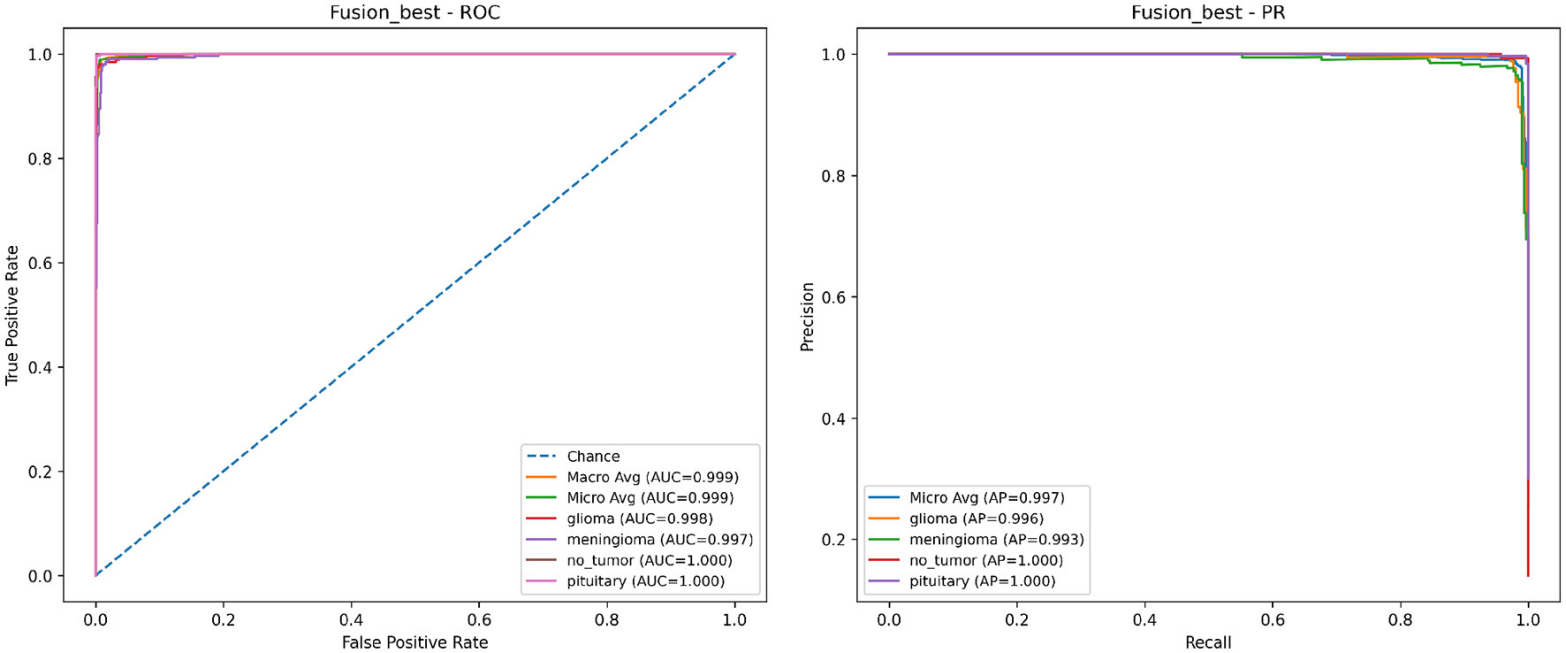
ROC **(left)** and Precision–Recall **(right)** curves for the proposed CNN–Swin fusion model under Cross-Dataset evaluation.

Receiver Operating Characteristic (ROC) and Precision–Recall (PR) curves provide threshold-independent insight into discrimination. Figure 8 illustrates class-wise ROC (left) and PR (right) curves for the proposed fusion model. Across tumor categories, the curves indicate strong separability under dataset shift, and PR behavior remains robust at high recall levels. This is particularly relevant in screening-oriented settings where false negatives can carry meaningful clinical risk.

### 5.4 Calibration and confidence reliability

Beyond discrimination, reliable confidence estimation is essential for safety-aware decision support: under dataset shift, overconfident incorrect predictions can obscure uncertainty and may mislead downstream interpretation. Table 5 reports Expected Calibration Error (ECE), Brier score, and Negative Log-Likelihood (NLL) for all models, reflecting *pre-calibration* confidence (i.e., without *post-hoc* temperature scaling).

**Table 5:** Calibration performance under Cross-Dataset evaluation.

Model	ECE	Brier	NLL
Swin Transformer	0.0289 ± 0.0098	0.0386 ± 0.0060	0.2885 ± 0.0450
CNN–Swin (additive fusion)	0.0217 ± 0.0074	0.0269 ± 0.0048	0.1723 ± 0.0321
CNN–Swin (concat fusion)	0.0104 ± 0.0038	0.0079 ± 0.0024	0.0612 ± 0.0185
ResNet50	0.0061 ± 0.0021	0.0036 ± 0.0010	0.0340 ± 0.0166
**CNN–Swin with HMSF (ours)**	**0.0041 ± 0.0022**	**0.0028 ± 0.0011**	**0.0277 ± 0.0100**

Lower values indicate better calibration. Bold values indicate best-performing results across models.

The standalone Swin Transformer exhibits severe miscalibration, with substantially higher ECE, Brier score, and NLL, consistent with overconfident errors under distribution shift. While the ResNet50 baseline demonstrates relatively stable calibration, the proposed fusion model achieves the best performance across all calibration metrics.

### 5.5 Effect size interpretation

Although absolute ECE differences may appear small in a low-error regime, the proposed fusion reduces ECE by approximately 33% relative to the strong CNN baseline (0.0061 → 0.0041) and by more than 85% relative to the transformer-only baseline (0.0289 → 0.0041). Similar relative reductions are observed for Brier score and NLL. These improvements indicate meaningfully better alignment between predicted confidence and empirical correctness under dataset shift.

**Figure 9:**
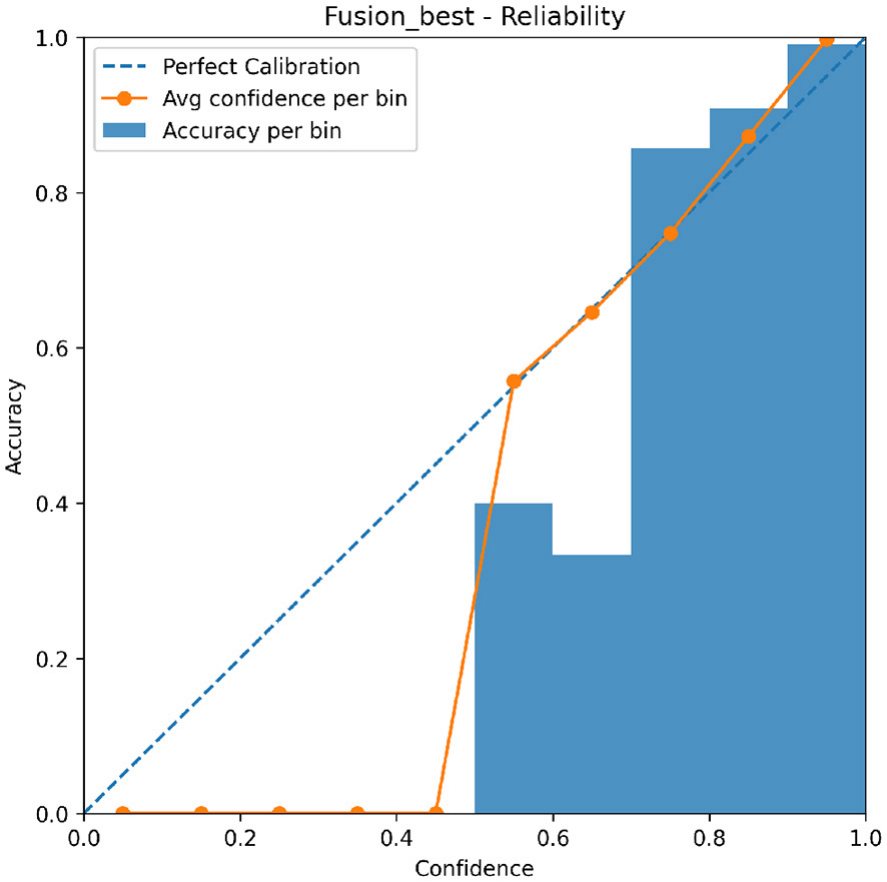
Reliability diagram comparing predicted confidence and empirical accuracy for the proposed fusion model under Cross-Dataset evaluation.

Figure 9 visualizes calibration behavior through a reliability diagram. The fusion model closely follows the diagonal, indicating improved alignment between predicted confidence and empirical accuracy.

### 5.6 Confidence distribution analysis

**Figure 10:**
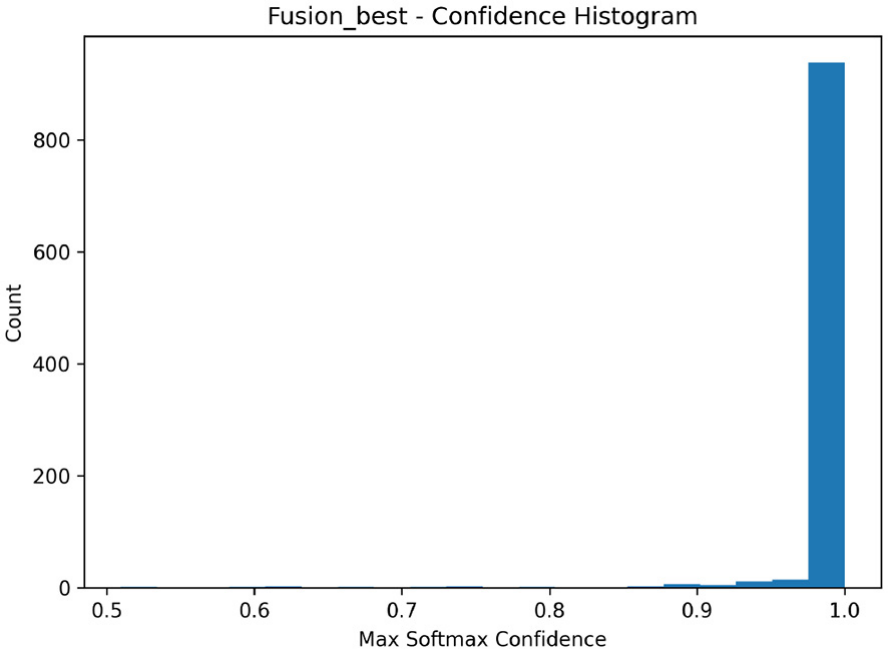
Histogram of predicted confidence values for correct and incorrect predictions under Cross-Dataset evaluation.

Figure 10 illustrates the distribution of predicted probabilities for correct and incorrect predictions. The fusion model assigns high confidence to correct predictions while exhibiting reduced confidence on misclassified or ambiguous cases. This behavior contrasts with the Swin Transformer, which tends to produce high-confidence errors under distribution shift.

### 5.7 Qualitative analysis and interpretability

This section provides qualitative evidence to complement the quantitative evaluation by visualizing spatial focus patterns used by the proposed fusion model. We report multi-scale attention maps as interpretability signals, illustrating how the model progressively refines its focus from coarse anatomical context to tumor-relevant regions.

### 5.8 Multi-scale attention visualization

**Figure 11:**
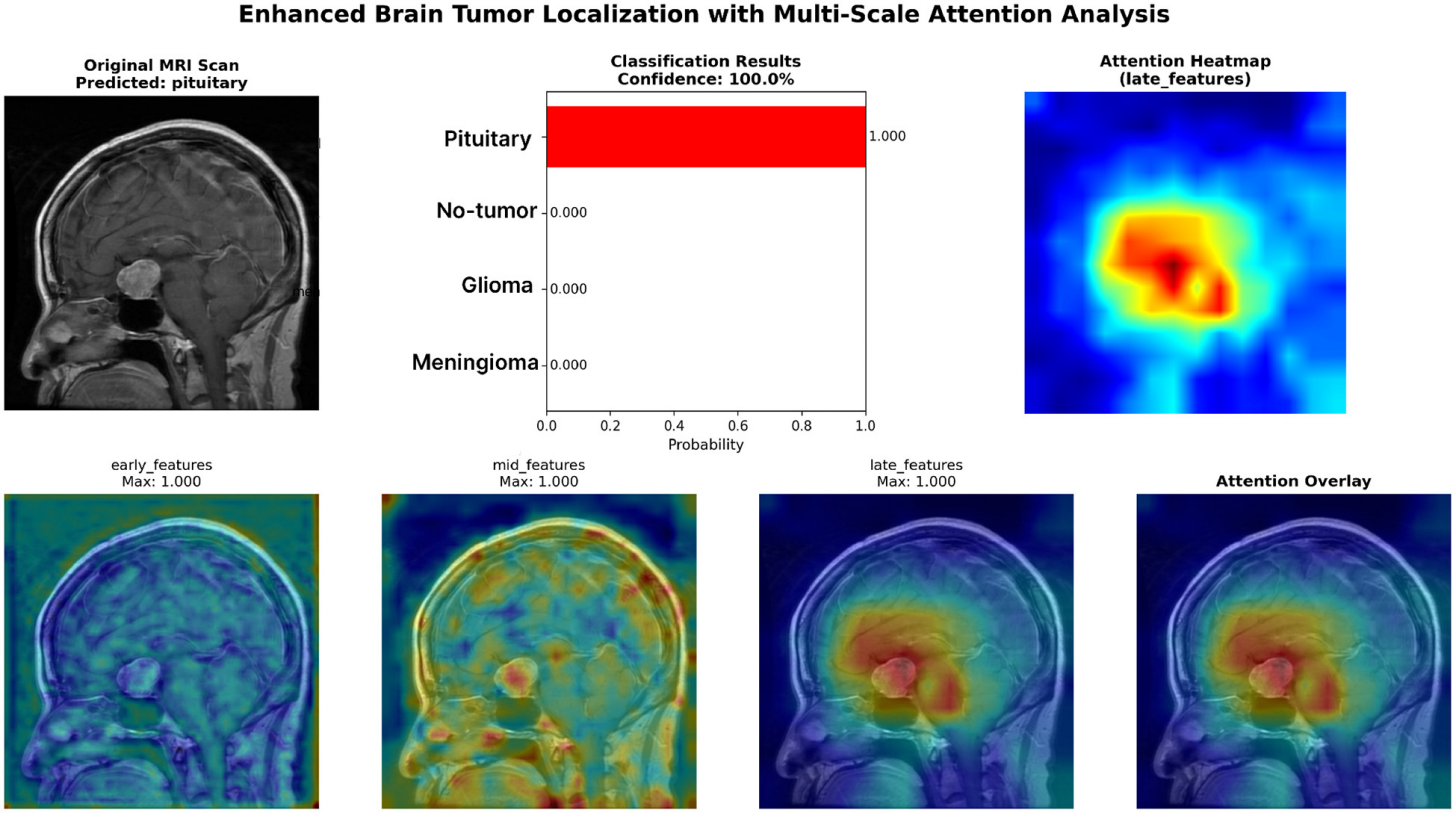
Multi-scale attention visualization of the CNN–Swin fusion model, illustrating progressive refinement from global context to tumor-relevant regions via weakly supervised attention cues.

Figure 11 shows representative multi-scale attention maps generated by the proposed fusion architecture. Early-stage maps highlight broad anatomical structures and candidate regions, mid-level maps increasingly concentrate around suspicious areas, and late-stage maps often produce compact responses concentrated in tumor-relevant regions, consistent with the predicted class.

The multi-scale attention patterns suggest complementary inductive biases: CNN features contribute spatial precision, while transformer features supply global context. Their cross-attention interaction yields attention maps that become more spatially concentrated at deeper stages, supporting class-consistent evidence aggregation. We emphasize that these maps serve as qualitative interpretability signals and do not constitute a segmentation output.

Although attention visualizations are useful for interpreting model behavior, they do not guarantee causal explanations. Additionally, without pixel-wise annotations, localization quality cannot be quantified as segmentation performance. We therefore report these maps as qualitative supporting evidence and recommend future work incorporating weakly supervised or fully supervised segmentation objectives for more rigorous localization validation.

## 6 Discussion

The experimental results demonstrate that hierarchical CNN-Transformer fusion can achieve a strong balance between discriminative performance and probabilistic reliability under strict Cross-Dataset evaluation. Standalone convolutional models benefit from locality-driven inductive biases that enhance robustness under moderate distribution shifts (Geirhos et al., 2020; Liu et al., 2022), which is consistent with the strong performance of the ResNet baseline in our experiments. However, their limited effective receptive fields may restrict the integration of global anatomical context, particularly when resolving ambiguous or spatially diffuse tumor patterns.

Transformer-based architectures such as the Swin Transformer are designed to capture long-range dependencies via self-attention (Liu et al., 2021). Yet, consistent with prior studies (Raghu et al., 2021; Ovadia et al., 2019), our Cross-Dataset results indicate that transformer-only models are more sensitive to dataset shift and prone to overconfident mispredictions. In safety-aware clinical settings, such confidence misalignment is critical: high-confidence errors may obscure uncertainty and compromise downstream decision-making even when overall accuracy appears competitive.

The proposed fusion framework mitigates these limitations through scale-aligned cross-attention, enabling convolutional features to query transformer representations in a structured manner. This asymmetric interaction preserves stable locality-driven evidence while adaptively incorporating global context. Unlike naive concatenation or additive aggregation, the proposed mechanism explicitly regulates feature interaction, preventing uncontrolled dominance of transformer representations under distribution shift. Empirically, this design yields improved Cross-Dataset classification performance alongside consistent gains in calibration metrics (ECE, Brier score, and NLL).

Although absolute calibration errors are small in low-error regimes, the relative improvements are substantial. HMSF reduces ECE by approximately 33% relative to a strong CNN baseline and by more than 85% relative to the transformer-only model, with comparable reductions in Brier score and NLL. These improvements reflect stronger alignment between predictive confidence and empirical correctness, which is essential when deploying models across scanners and institutions with heterogeneous acquisition characteristics.

Importantly, the reported improvements are obtained in a *pre-calibration* setting, without temperature scaling, ensembling, or *post-hoc* correction. This suggests that architectural design alone can meaningfully influence confidence reliability. While *post-hoc* calibration techniques remain valuable, our findings indicate that reliability can be embedded directly within the feature-interaction mechanism. Because the proposed fusion operates at a multi-scale representational level, the same principle is extensible to other brain MRI tasks, including lesion grading, subtype stratification, and weakly supervised localization under multi-center heterogeneity.

## 7 Conclusion and future work

This work introduced a calibration-aware hierarchical CNN-Transformer fusion framework designed as a robust brain MRI analysis method under dataset shift. By integrating convolutional inductive biases with transformer-based global context modeling through scale-aligned cross-attention, the proposed approach achieves competitive discriminative performance while substantially improving probabilistic reliability in strict Cross-Dataset evaluation settings.

Extensive experiments demonstrate that the proposed fusion design not only maintains high classification accuracy but also consistently improves *pre-calibration* confidence behavior, as evidenced by reduced Expected Calibration Error (ECE), Brier score, and Negative Log-Likelihood (NLL). These findings indicate that architectural fusion choices can directly influence the quality of predictive confidence, complementing *post-hoc* calibration and ensemble-based uncertainty estimation techniques rather than replacing them. Improved alignment between predictive confidence and empirical correctness is a necessary condition for safety-aware decision support in brain imaging applications (Esteva et al., 2021).

By evaluating all models exclusively under a train-on-one-dataset, test-on-another protocol, this study provides a deployment-relevant assessment of generalization and confidence reliability in the presence of dataset shift. The results suggest that hierarchical CNN-Transformer fusion represents a promising methodological direction for brain MRI analysis tasks that require both robustness and trustworthy probabilistic outputs.

Several directions for future work naturally emerge. First, while the current study focuses on 2D slice-based analysis, extending the proposed fusion framework to volumetric 3D MRI represents an important next step. Incorporating 3D convolutional encoders and volumetric transformers may further improve anatomical consistency and spatial reasoning (Hatamizadeh et al., 2022a). Second, integrating test-time adaptation or online calibration strategies could enhance robustness under more severe or evolving distribution shifts, building on recent advances in self-supervised and entropy-based adaptation methods (Wang D. et al., 2021). Finally, uncertainty-aware deployment strategies, such as selective prediction or human-in-the-loop workflows, offer a practical pathway for incorporating the proposed framework into real-world clinical and large-scale neuroscience research pipelines while maintaining appropriate safety and oversight.

## Data Availability

The datasets analyzed in this study are publicly available and were obtained from open-access repositories. The first dataset is the BRISC (Brain Tumor Image Segmentation and Classification) dataset, which provides annotated brain MRI scans for tumor classification and related research tasks. The dataset is publicly available via Figshare at https://doi.org/10.6084/m9.figshare.30533120 and is described in the associated publication by Fateh et al. The data are released for research use and include expert-provided diagnostic labels. The second dataset is the BT-MRI four-class brain tumor classification dataset, which contains contrast-enhanced brain MRI slices categorized into glioma, meningioma, pituitary tumor, and no-tumor classes. This dataset is publicly available on Kaggle at https://www.kaggle.com/datasets/mohamadabouali1/mri-brain-tumor-dataset-4-class-7023-images. In this study, one dataset is used exclusively for training and the other exclusively for testing, following a strict train-on-one, test-on-another cross-dataset evaluation protocol. All preprocessing steps, dataset partitions, and experimental configurations are described in the manuscript to support reproducibility.
